# Bridging the gap: insights into sensorimotor deficits in NMDA receptor antibody encephalitis

**DOI:** 10.1172/JCI188251

**Published:** 2025-03-03

**Authors:** Puneet Opal, Geoffrey T. Swanson

**Affiliations:** 1 Denning Ataxia Center, Davee Department of Neurology and Department of Cell and Developmental Biology, Northwestern University Feinberg School of Medicine, Chicago, Illinois,USA.; 2 Department of Pharmacology, Northwestern University Feinberg School of Medicine, Chicago, Illinois, USA.; 3 Department of Neurobiology, Weinberg College of Arts and Sciences, Northwestern University, Evanston, Illinois, USA.

## Abstract

*N*-methyl-d-aspartate (NMDA) receptor–mediated autoimmune encephalitis (NMDAR-AE) is the most common cause of autoimmune encephalitis, especially in children and young adults. The disorder is caused by antibodies directed against the GluN1 protein, an obligatory constituent of NMDA receptors, which are key signaling molecules in brain development, learning and memory, and executive function. The manuscript by Zhou et al. offers key insights into aberrant development of cortical pathways that may underly persistent sensorimotor deficits associated with this encephalitis in a newly generated mouse model. This study convincingly links transient exposure to a patient-derived anti-GluN1 mAb during a critical developmental period to lasting disruptions in interhemispheric connectivity through callosal projections. These findings provide insight into the impact of a prevalent autoimmune disorder on fundamental aspects of brain development and establish a model system that could be further employed to probe other aspects of NMDAR-AE pathogenesis.

## The pathology of NMDAR-AE

*N*-methyl-d-aspartate (NMDA) receptor–mediated autoimmune encephalitis (NMDAR-AE) is a severe autoimmune condition with diverse manifestations, including behavioral and psychiatric symptoms, sensorimotor deficits, movement disorders, and seizures. First described in women with ovarian teratomas (1), the disorder was later documented in a broader population, including children and young adults (2, 3). The condition, with its bewildering spectrum of symptoms, came to widespread public attention following the publication and subsequent film adaptation of *Brain of Fire: My Month of Madne*ss, a poignant story about the NMDAR-AE experience told from a patient’s perspective (4).

The pathology of NMDAR-AE arises from antibodies targeting NMDA receptor subunits, particularly GluN1 (5). NMDA receptors are membrane-spanning ion channels activated by the neurotransmitters glutamate and glycine and are critical mediators of many forms of learning and memory. These receptors are essential for synaptic plasticity, learning, memory, and numerous developmental processes such as axonal arborization, dendritic maturation, and synaptogenesis (6). Disruption of NMDA receptor function — as occurs in NMDAR-AE — results in cognitive and neurological impairments of varying severity and duration.

Research on NMDAR-AE has primarily focused on the acute effects of anti-NMDAR antibodies on glutamatergic circuits that relates to the clinical manifestations of the disorder — and the efficacy of immunotherapy as a way to mitigate the pathogenic role of these antibodies (7–9). However, clinicians have observed that therapeutic interventions often fail to fully resolve deficits after prolonged antibody exposure. Moreover, in the rare cases of transplacental transfer of antibodies to the fetus, brain development is substantially impacted (8). These observations led to the conclusion that persistent “hard-wired” alterations occur with exposure to anti-GluN1 antibodies, an idea which has been supported in mouse models of gestational exposure to anti-NMDAR antibodies that caused pathological changes in brain morphology and deficits in behavior (10)—indeed, many of these changes manifest well after the mice reach maturity (11).

## A developmental mouse model of NMDAR-AE

The developmental basis for those phenotypes were not determined, but could provide crucial insights for devising long-term treatment strategies in NMDAR-AE patients. In this issue of the *JCI*, Zhou et al. (12) address this knowledge gap by creating a mouse model to study the lasting effects of developmental exposure to anti-NMDAR antibodies. This study builds on the authors’ previous work in which they observed sensorimotor deficits and altered cortical development after genetic deletion of *Grin1*, the gene encoding GluN1, or by immune targeting of GluN1 protein with commercially available antibodies (13). These results logically led to the hypothesis that bonafide disease-causing antibodies generated by patients with NMDAR-AE would also have similar deleterious effects on brain development, thereby providing an explanation for persistence of the disorder despite removal of the offending antibodies in patients.

**Figure 1:**
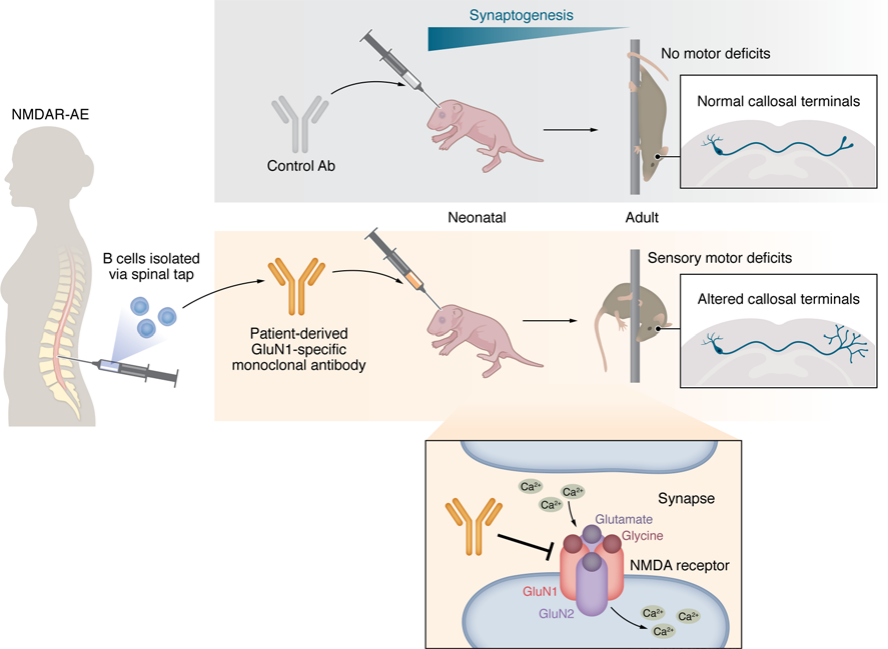
Anti-GluN1 antibody exposure at an early stage of cortical development affects brain function into adulthood. Zhou and colleagues produced mAbs against GluN1 after isolating B cells from a patient with NMDAR-AE. Administration of mAb3^[GluN1]^ to mice from postnatal day 3 to 12 resulted in long-lasting sensorimotor effects. Critical developmental events during this period involve interhemispheric connectivity through callosal projections and synaptogenesis. Correspondingly, young mice showed morphological changes, including increased axon branching at terminals, that persisted with age (12).

To test this idea, the authors carried out a rigorous, multilevel analysis of cellular, network, and behavioral parameters in mice following unilateral intraventricular administration of an anti-GluN1 monoclonal antibody (mAb3^[GluN1]^) derived from a patient with NMDAR-AE. The antibody was injected into neonatal mice from postnatal day 3 to 12, mimicking an early onset autoimmune response in patients. The authors then sought to understand the acute and long-term consequences of that insult on acute NMDA receptor signaling, cortical innervation across and within hemispheres, network excitability, functional connectivity, and motor performance (Figure 1) (12).

Zhou et al. focused their efforts on analysis of projections from the primary somatosensory cortex (S1) through the callosum to the contralateral hemisphere because this circuit is critical for bilateral sensorimotor integration (12). They demonstrated that cortical NMDA receptor signaling at synapses formed by these projections was reduced in juvenile mice, validating the treatment as a model for NMDAR-AE. In young adult mice, callosal projections to the mAb3^[GluN1)^ antibody–treated hemisphere terminated in a wider area of the somatosensory cortex and were more numerous compared to those in mice that received control antibody. As predicted by their model, mice treated with the GluN1 antibody developed gross motor skills, but exhibited prolonged deficits in a variety of tasks requiring fine sensorimotor integration. Functional deficits between somatosensory and motor cortices in the test mice were detected using multielectrode array recordings. Notably, intrahemispheric connectivity between the primary sensory and motor cortices remained intact, suggesting that the observed deficits stemmed specifically from callosal disruptions.

The authors concluded the study by examining whether the mAb3^[GluN1]^-induced morphological changes observed in young adult mice persist with age. They found that the excessive callosal projections had been winnowed down to a pattern resembling that in the control group, but other morphological alterations, including increased branching of the interhemispheric projections, remained and increased complexity at a finer level of the interhemispheric axons. Surprisingly, the effect of mAb3^[GluN1]^ was greater in male mice compared with females, correlating with a similar bias in performance in some behavioral tasks. Enhanced cortical excitability in the treated mice provided further evidence for the persistent impact of perinatal exposure to the human antibody (12).

## Conclusion and implications

In summary, the report by Zhou et al. connects the molecular effects of an anti-GluN1 antibody at an early stage of cortical development to change in brain function into adulthood (12). The narrow focus of the study on sensorimotor development and performance leaves open the possibility that development of other cortical systems is similarly disrupted. A further challenge lies in the correlative nature of the observations — a causative link between altered callosal projects and the higher-level measure of function remains to be established. The full extent of phenotypic differences in male and female mice exposed to mAb3^[GluN1]^ also will be of interest, particularly considering the broader literature on sex differences in neurodevelopmental disorders, and warrants further investigation. The authors have established a tractable model system to explore the cellular and systems basis of dysfunction in NMDAR-AE in these and other future studies (12).

Zhou et al.’s work underscores the importance of early diagnosis and intervention in pediatric NMDAR-AE to prevent irreversible developmental changes. The study also raises the possibility that similar mechanisms may underlie persistent deficits in autoimmune conditions caused by less common anti-glutamate receptor antibodies or those targeting other membrane proteins key to brain development (14–16). This research also highlights the profound and lasting impact of early developmental disruptions, emphasizing the need for timely intervention and the exploration of strategies to mitigate long-term consequences in both neurodevelopmental and neurodegenerative disorders (12, 17, 18).

## Version 1 03/03/2025

Electronic publication
